# 

**Published:** 1871-08

**Authors:** 


*Southern Dental Association.*—The following is a list of the Officers and Committees of the Southern Dental Association, until the next Annual Meeting of that body, to be held in the city of Richmond, Va., the last Tuesday in July 1872, commencing at 10 o'clock A. M.

It is sincerely hoped and expected, that the members of the following Committees will correspond one with another, so as to act in concert, and furnish a report on each subject, and thereby make our next meeting one of the most interesting and instructive ever held, as it is expected to be the largest.

OFFICERS.

*President*, Dr. F. Y. Clark, Georgia; 1*st Vice President*, Dr. J. B. Patrick, S. C.; 2*nd Vice President*, Dr. H. M. Grant, Va.; 3*d Vice President*, Dr. E. Floyd, N. C.; *Corresponding Sec'y*, Dr. A. C. Ford, Georgia; *Recording Sec'y*, Dr. O. J, Bond, S. C.; *Treasurer*, Dr. W. G. Redman, Ky.

*Executive Committee*, Drs. H. M. Grant, Va.; Jas. F. Thompson, Va.; L. G. Holland, Georgia; E. A. Herman, Tenn., and T. T. Moore, South Carolina.

*Committee on Membership*, Drs. James F. Thompson, Va.; J. B. Patrick, S. C.; and H. C. Jones, Virginia.

*Committee on Publication*, Drs. Samuel A. White, E. M. Allen, Georgia, and F. J. S. Gorgas, Md.

*Committee on Dental Education*, Drs. J. P. H. Brown, Georgia; Geo. H. Winkler, S. C., and John G. Angell, Louisiana.

*Committee on Physiology and Surgery*, Drs. W. T. Arrington, Tenn.; W. H. Atkinson, N. Y., and James S. Knapp, Louisiana.

*Committee on Histology and Microscopy*, Drs. S. P. Cutler, Louisiana: G. T. Barker, Penn., and William Reynolds, South Carolina.

*Committee on Dental Chemistry*, Drs. B. F. Arrington. N. C.; W. H. Morgan, Tenn., and J. G. McAuley, Alabama.

*Committee on Dental Therapeutics*, Drs. N. W. Kingsley, N. Y.; W. H. Burr, Georgia, and Samuel Rambo, Alabama.

*Committee on Operative Dentistry*, Drs. E. A. Herman, Tenn.; A. C. Ford, Georgia, and W. H. Shadoan, Kentucky.

*Committee on Mechanical Dentistry*, Drs. B. A, Rodrigues, S. C.; J. H. Royal, Georgia, and G. F. Wright, South Carolina.

*Committee on Dental Literature*, Drs. F. J. S. Gorgas, Md.; W. C. Wardlaw, S. C., and E. Floyd, North Carolina.

*Committee on Voluntary Essays*, Drs. H. A. Lowrance, Ga.; Samuel A. White, Georgia, and S. J. Cobb. Tennessee.

---

